# The tryptophan-kynurenine pathway in cardiovascular diseases: mechanistic insights and therapeutic opportunities

**DOI:** 10.3389/fcvm.2026.1726522

**Published:** 2026-03-11

**Authors:** Wei Chen, Min Shui, Zhen Wei, Weiyan Gao, Xin Liu, Qian Lei

**Affiliations:** 1 Department of Anesthesiology, Sichuan Provincial People’s Hospital, School of Medicine, University of Electronic Science and Technology of China, Chengdu, China; 2 Department of Anesthesiology, Sichuan Academy of Medical Sciences & Sichuan Provincial People’s Hospital, School of Medicine, University of Electronic Science and Technology of China, Chengdu, China; 3 Department of Anesthesiology, Affiliated Hospital of Southwest Medical University, Luzhou, Sichuan, China; 4 Laboratory of Mitochondria and Metabolism, National-Local Joint Engineering Research Centre of Translational Medicine of Anesthesiology, Department of Anesthesiology, West China Hospital, Sichuan University, Chengdu, Sichuan, China

**Keywords:** cardiovascular diseases, kynurenine pathway, metabolomics, tryptophan metabolism, treatment

## Abstract

Cardiovascular diseases (CVDs) are the leading cause of death worldwide, making them crucial to further explore their mechanisms. Beyond traditional risk factors, disturbances in tryptophan metabolism, particularly the imbalance in the kynurenine pathway (KP) which accounts for over 95% of metabolic flux—have garnered significant attention in cardiovascular research. In the human body, tryptophan is primarily metabolized through the KP. This process is catalyzed by key enzymes, indoleamine 2,3-dioxygenase and tryptophan 2,3-dioxygenase, which convert tryptophan into kynurenine and further downstream metabolites such as kynurenic acid and 3-hydroxykynurenine. Studies have shown that levels of multiple key metabolites in KP are dysregulated in patients with CVDs, and by participating in processes such as immune activation, inflammatory responses, reactive oxygen species production, and endothelial dysfunction, and they play a complex role in mediating the pathophysiology of various CVDs, including heart failure, atherosclerosis, and hypertension. This review will systematically outline the physiology of tryptophan metabolism and the KP, summarize how key enzymes and metabolites regulate CVDs, and explore their potential as novel biomarkers for early diagnosis and prognosis and as therapeutic targets. Additionally, the review will discuss the future applications of metabolomics and artificial intelligence in the diagnosis of CVDs and the development of new therapeutics, aiming to provide new perspectives for the prevention and treatment of CVDs.

## 1 Introduction

Cardiovascular diseases (CVDs) are the leading cause of death worldwide, responsible for approximately 20 million deaths annually. Their prevalence has nearly doubled over the past two decades, posing a severe threat to global health (1, 2). In the European Union, CVDs account for 11% of the total healthcare expenditure, representing a substantial societal burden (3). The pathogenesis of CVDs is complex and multifactorial. Beyond the well-established traditional risk factors such as hyperlipidemia, diabetes mellitus, and smoking, recent studies have highlighted the close association between metabolic disorders—particularly those involving tryptophan metabolism—and CVDs, offering a novel perspective for understanding their pathophysiology (4).

**Figure 1:**
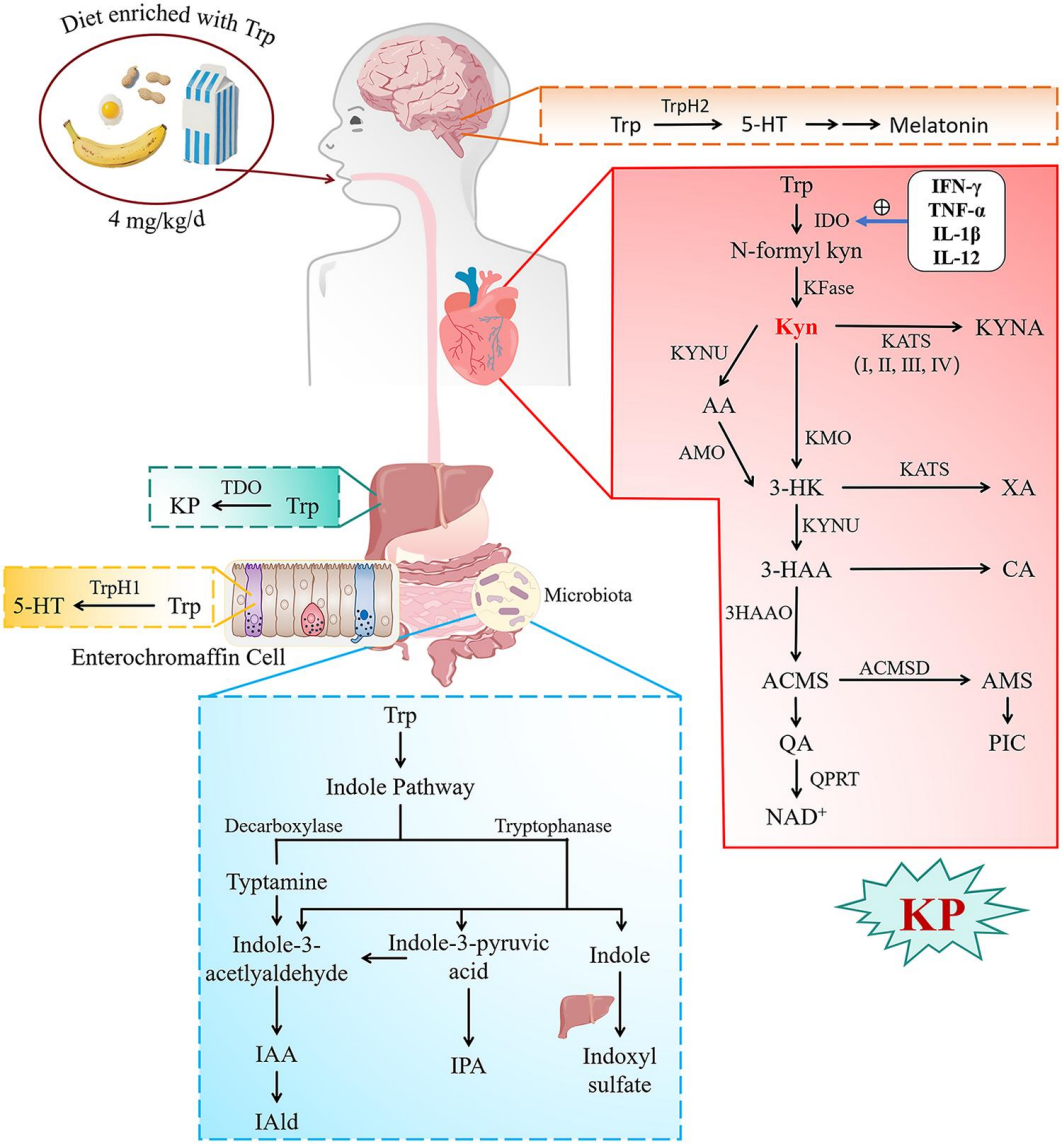
The figure shows the metabolites and enzymes of the three tryptophan metabolic pathways in the human body (see text for details). TryptophanH 1 and 2, Tryptophan Hydroxylase 1 and 2, 5-HT, 5-hydroxytryptamine; IPA, Indole-3propionic acid; IAA, Indole acetic acid; IAld, Indole-3-aldehyde; Trp, Tryptophan; N-formyl KYN, N-formyl-Kynurenine; KYNA, Kynurenic acid; AA, anthranilic acid; 3-HAA, 3-Hydroxyanthranilic acid; 3-HK, 3-Hydroxykynurenine; XA, xanthurenic acid; CA, cinnabarinic acid; ACMS, 2-amino-3-carboxymuconate semialdehyde; AMS, 2-aminomuconic-6-semialdehyde; PIC, picolinic acid; QA, quinolinic acid; NAD+, Nicotinamide Adenine Dinucleotide; IDO1 and 2, Indoleamine 2,3-Dioxygenase 1 and 2; TDO, Tryptophan 2,3-dioxygenase, KATs, Kynurenine Aminotransferases; KMO, Kynurenine-3-Monooxygenase; KYNU, Kynureninase; AMO, anthranilate 3-monooxygenase; 3-HAAO, 3-hydroxyanthranilic acid oxygenase; ACMSD, 2-amino-3-carboxymuconic acid semialdehyde decarboxylase' QPRT, quinolinate phosphoribosyl transferase; IFN-*γ*, Interferon-*γ*; TNF-α, tumor necrosis factor-α; IL-1β, interleukin-1 *β*.

Tryptophan (Trp) is an essential aromatic amino acid that cannot be synthesized endogenously and must be acquired through the diet, with common sources including eggs, grains, dairy products, and bananas. The World Health Organization (WHO) recommends a daily intake of 4 mg/kg (5). Trp is not only an important raw material for protein synthesis, but also a precursor of many important bioactive molecules. Trp was first identified as an important biochemical precursor of serotonin and melatonin in the brain, research on Trp was predominantly centered on neurological disorders, such as neurodegenerative diseases and depression (6, 7). Subsequent elucidation of its metabolic pathways has revealed that Trp is catabolized through three pathways (Figure 1). The majority (95%) of Trp is metabolized via the KP, whereas 4%–6% is diverted to the indole pathway, where the gut microbiota produces indole metabolites (8). Only 1%–2% of Trp enters the serotonin/melatonin pathway (9). Due to the discovery of KP and indole pathways, the influence of Trp has been extended to a variety of pathological and physiological processes such as inflammatory response, oxidative stress, endothelial dysfunction and intestinal homeostasis (10).

Recent epidemiological evidence further indicates that lower circulating level of Trp is significantly associated with an elevated risk of CVDs (11). Moreover, altered levels of kynurenine (KYN) and its derivatives have been observed in various CVDs, including atherosclerosis, heart failure, and myocardial ischemia (12–14). This indicates that the imbalance of the tryptophan-Kynurenine (Trp-KYN) pathway plays an important role in the occurrence and development of CVDs. Oxidative stress and inflammation are considered central mechanisms in CVDs, and the accelerated metabolism of Trp-KYN pathway is closely related to inflammatory activation, immune dysregulation and oxidative stress which may further drive disease progression (15). However, its specific mechanism has not been fully elucidated. How KP participates in the process of CVDs by regulating immunity, inflammation and endothelial function still need to be verified by more functional experiments. In addition, there are inconsistencies in the results of existing studies, such as the trend of metabolites of KP in different CVDs.

Therefore, this article systematically reviews the role of tryptophan metabolic pathway and its metabolites in CVDs, focusing on the molecular interaction mechanism of KP in atherosclerosis, heart failure, hypertension and other diseases. It also discusses the clinical value as a new biomarker and potential therapeutic target for early diagnosis and prognosis evaluation. Finally, by integrating emerging technologies such as metabolomics and artificial intelligence, the application prospect of this pathway in the diagnosis, prognosis and individualized treatment of CVDs is prospected, in order to provide new theoretical basis and strategic support for the prevention and treatment of CVDs.

## 2 Physiological basis of tryptophan metabolism

To better understand the role of tryptophan metabolism in various CVDs, it is necessary to clarify its metabolic pathways and key products to lay the foundation for further understanding.

### 2.1 Kynurenine pathway

The discovery of tryptophan metabolism and related substances unfolded in unexpected sequences. Kynurenic acid (KYNA) was found in urine of dog as early as 1,853 by Justus von Liebig (16), while Trp was not identified as an essential amino acid until 1,901 (17), and it was confirmed as the precursor of KYNA in 1,904 (18). Despite these initial findings, it was not until the mid to late 20th century that the complete Trp-KYN pathway was gradually elucidated. First, Trp is metabolized by the rate-limiting enzyme tryptophan-2,3-dioxygenase (TDO) in the liver or indoleamine-2,3-dioxygenase (IDO) outside the liver to form N-formyl-Kynurenine, which is then rapidly converted to KYN by formamidase. This is the central product of the KP (19). Next, KYN is catalyzed by three different enzymes to form three metabolic branches (Figure 1). The first branch involves the conversion of KYN to KYNA by kynurenic acid transaminase (KATs) (20). In the second branch, Kynureninase (KYNU) converts KYN to anthranilic acid (AA), which is further metabolized by anthranilate 3-monooxygenase (AMO) to 3-hydroxyanthranilic acid (3-HAA) (21). In the third branch, Kynurenine monooxygenase (KMO) produces 3-hydroxkyurenine (3-HK), which is further metabolized by KYNU to 3-HAA (22). 3-HK can also be metabolized by KATs to form xanthurenic acid (XA) or auto-oxidized. Further down, 3-HAA is converted by 3-hydroxyanthranilic acid oxygenase (3-HAAO) to 2-amino-3-carboxymuconate semialdehyde (ACMS) or suffers auto-oxidation to form cinnabarinic acid (CA) (23). ACMS is unstable and spontaneously generates quinolinic acid (QA) after non-enzymatic cyclization. However, ACMS may be further metabolized by 2-amino-3-carboxymuconic acid semialdehyde decarboxylase (ACMSD) to form 2-aminomuconic-6-semialdehyde (AMS). The latter can spontaneously form neuroprotective picolinic acid (PIC) (24). Quinolinate phosphoribosyl transferase (QPRT) converts QA to nicotinamide mononucleotide, whose further transformations lead to the formation of NAD+ (25). The entire metabolic process is completed.

#### 2.1.1 Key enzymes: TDO and IDO

TDO and IDO are the first rate-limiting enzymes of the KP and perform the same metabolism. Under steady-state conditions, extrahepatic KP accounts for the smallest proportion (<2%); however, under inflammatory conditions, IDO activity exceeds that of hepatic TDO, and its proportion becomes significant (26).

IDO is a cytoplasmic monomer reductase that is widely expressed in various cells and tissues outside the liver, including monocytes/macrophages, dendritic cells, lymphocytes, intestinal epithelial cells, etc (27). IDO is normally expressed at low levels; however, its activity is upregulated by inflammatory factors during inflammation, primarily through the JAK/STAT1 pathway induced by Interferon-*γ* (IFN-*γ*) (Figure 1). This is because the IFN-*γ*-IDO axis is a genetically conserved mechanism that limits microbial growth and prevents potentially harmful, excessive inflammatory responses in the host (28). In addition, IDO can also rely on inflammatory factors such as tumor necrosis factor-α (TNF-α), interleukin-1 β (IL-1β), and IL-12 for synergistic activation (29). This regulation helps IDO exert immunosuppression by consuming tryptophan under pathological conditions. Now, the biological effects of IDO extend beyond its role in regulating immune responses and are widely involved in the onset and development of CVDs.

TDO is a tetrameric heme enzyme that is highly expressed in the liver cells of mammals. It is upregulated and maintained by tryptophan, glucocorticoids, and the lipid metabolite prostaglandin E2 (PGE 2) (30). It participates in metabolic balance by maintaining tryptophan homeostasis and regulating NAD^+^ precursor levels, accounting for 80%–95% of dietary tryptophan metabolism (31). Recent studies have found that TDO plays a key role in human umbilical vein endothelial cells (HUVECs) and endothelial cell colony-forming cells (ECFCs), promoting *in vitro* proliferation and capillary morphogenesis, and its role in angiogenesis challenges the established notions of immune regulation (32).

#### 2.1.2 Fundamental biological functions of KP

For a long time, metabolites of KP have been classified as common metabolic by-products. However, it was with advances in molecular and functional research that their roles in key physiological and pathological processes—such as immune regulation, neural function, inflammation, and oxidative stress—gradually came to be recognized (Table 1). The following is a brief introduction.

**Table 1:** Brief description of the effects of selected TRP–KYN pathway metabolites.

Metabolites	Biological functions	Reference
KYN	Immunosuppression, antioxidant, promotes oxidation	(33–35)
KYNA	Immunosuppression, neuroprotective, antioxidant, promotes oxidation	(36–38)
QA	Excitotoxic, gliotoxic, promotes oxidation	(39, 40)
3-HK	Neurotoxic, antioxidant, generates free radicals	(41–43)
PIC	Immunosuppression, metal ionophore, antioxidant	(44, 45)
NAD+	Antioxidant	(46)

Metabolites of KP have immunosuppressive functions and play an important role in immune regulation. For example, by activating the aryl hydrocarbon receptor (AhR) in naive T cells, KYN and KYNA under physiological conditions inhibit effector-T-cell proliferation, up-regulate FoxP3, and drive differentiation of regulatory T cells (33, 41). This process helps to prevent excessive immune response, thereby avoiding the occurrence of autoimmune diseases and chronic inflammation. It is also an important mechanism for the formation of immune tolerance, and can even prolong the survival time of corneal allografts. However, the immunosuppressive microenvironment formed by KYN is also a key mechanism involved in the immune evasion of various cancers (47). Unlike KYN, *in vitro* experiments have shown that PA does not disrupt cell viability and does not interfere with classical pathways such as MAPK, mTOR, and NF-*κ*B. Instead, it inhibits T cell proliferation and metabolism in a concentration-dependent manner by specifically reducing the phosphorylation at the Ser62 site of the c-Myc protein, demonstrating precise proliferation inhibition rather than total immune paralysis (44). In addition, other derivatives of KYN pathway (3-HK,3-HAA etc.) also have immunomodulatory effects (48–50). Due to limited space, this review can be referred to regarding immune effects, which will not be presented in detail (51). The specific immune response of KP in CVDs will be described in detail later.

Metabolites of KP play pivotal roles in the central nervous system, and their dysregulation is broadly implicated in the pathophysiology of neurological and psychiatric disorders. The neurotoxic arm of the pathway is represented by QA and 3-HK. Under pathological conditions, QA rises to 500–1,200 nM, over-activates N-methyl-D-aspartic acid receptors (NMDARs), triggers neuronal excitotoxicity and astrocytic Ca²^+^ overload, and impairs astrocytic glutamate uptake, leading to extracellular glutamate accumulation (39). QA also promotes reactive oxygen species generation and lipid peroxidation, inflicting oxidative damage, and may destabilize the cytoskeleton via aberrant phosphorylation, further aggravating neurotoxicity (40). In various disease contexts, 3-HK (>0.3 μM in brain) mediates neurotoxicity through multiple mechanisms, including mitochondrial dysfunction, abnormal protein aggregation, and DNA damage, synergistically amplifying oxidative stress together with QA (52). Clinical studies demonstrate significantly elevated QA and 3-HK levels in the brains of Alzheimer's disease (AD) and Parkinson's disease (PD) patients, with the magnitude correlating with disease severity (39, 53, 54). Conversely, KYNA constitutes the neuroprotective branch. As an endogenous antagonist of NMDARs, KYNA blocks glutamate excitotoxicity at nanomolar concentrations. It also exerts anti-inflammatory effects via G-protein coupled receptor 35 (GPR35) activation and directly scavenges free radicals (36). In AD、PD and related neurodegenerative diseases, brain KYNA is frequently decreased (55, 56), and this loss of protection further amplifies neuronal injury. Thus, an imbalance of KP metabolism—characterized by increased neurotoxic metabolites (QA, 3-HK) and decreased neuroprotective KYNA—exposes the nervous system to persistent excitotoxicity, oxidative stress, and neuroinflammation, creating a self-reinforcing vicious cycle. Understanding the dual nature of the KP pathway and re-establishing its equilibrium, either by inhibiting the toxic branch or enhancing the protective branch, may restore CNS homeostasis and offer novel therapeutic avenues. However, its similar function in the heart needs to be further verified in cardiovascular models.

In the context of oxidative stress, metabolites of KP play complex and dynamic dual regulatory roles. Their ultimate effect—whether antioxidative or pro-oxidative—is not fixed but depends on a dynamic balance of local concentration, cellular microenvironment, and metabolic status. Metabolites of KP generally exhibit concentration-dependent effects. At physiological or relatively low concentrations (e.g., serum KYN ∼1–3 µM, serum KYNA ∼5–30 nM, or brain 3-HK ∼0.08–0.3 µM), they can effectively scavenge various reactive oxygen/nitrogen species such as hydroxyl radicals and peroxynitrite, reduce lipid peroxidation levels, and demonstrate significant antioxidant activity (37–42). However, under pathological conditions, when metabolic dysregulation leads to abnormally high accumulation of these metabolites, their function often reverses, turning them into pro-oxidants. KYN and KYNA, at excessive concentrations, disrupt mitochondrial function and induce the overproduction of reactive oxygen species (ROS) (35, 57). In neurodegenerative diseases, abnormally elevated 3-HK can directly damage the mitochondrial electron transport chain, triggering a burst of ROS that results in severe oxidative damage to lipids, proteins, and DNA, ultimately driving neuronal death (43). The pro-oxidative effect of QA is primarily achieved indirectly through excitotoxicity mediated by overactivation of NMDA receptors and the induction of mitochondrial dysfunction (40). In contrast, the end product of the KP, NAD+, has a relatively unidirectional function. As the core coenzyme of mitochondrial energy metabolism, NAD^+^ maintains redox homeostasis by regulating the NAD^+^/NADH ratio. In cardiovascular diseases, NAD^+^ depletion is associated with impaired cardiac energy metabolism and exacerbated oxidative stress; preclinical evidence demonstrates that restoring NAD^+^ levels can ameliorate mitochondrial function and delay disease progression, though human validation requires further investigation (58, 59). Notably, due to the lack of a complete enzyme system required for *de novo* NAD^+^ synthesis via the KP, the heart primarily relies on precursors such as nicotinamide to maintain NAD^+^ homeostasis through the salvage pathway, suggesting that NAD^+^ produced by the KP contributes limitedly to the heart (60). However, the role of NAD^+^ in cardiovascular diseases can be found in this review (61). PIC, an isomer of nicotinic acid, functions as a specific chelator for zinc, iron, and chromium ions. Studies have demonstrated that its complex, chromium picolinate, can mitigate chronic exercise-induced oxidative stress by activating the glutathione antioxidant system; in cardiovascular disease models, this complex has further been shown to improve coronary perfusion, attenuate myocardial oxidative damage, and enhance cardiac function in hypoxic rats (45, 62). However, PIC primarily acts as a metal ion carrier, and there is a paucity of studies investigating the role of PIC alone in CVDs, with limited direct evidence supporting its cardioprotective effects. Future studies are warranted to elucidate the independent mechanisms of PIC in cardiovascular regulation.

In summary, metabolites of KP constitute a finely tuned regulatory network for oxidative stress. Under physiological conditions, they serve as important components of the antioxidative defense system, whereas under pathological conditions, they can transform into potent drivers of oxidative damage.

### 2.2 5-hydroxytryptamine metabolic pathway

5-hydroxytryptamine (5-HT) is an important monoamine neurotransmitter, the synthesis of which is mainly concentrated in the intestine and nervous tissue. In the intestine, dietary tryptophan is catalyzed by tryptophan hydroxylase 1 (TryptophanH1) in enterochromaffin cells to form 5-hydroxytryptophan (5-HTP), which is then decarboxylated by aromatic L-α-amino acid decarboxylase (AADC) in the presence of the cofactor PLP to form 5-HT. This process accounts for 90% of the body's total 5-HT. The central nervous system relies on TryptophanH2 to independently synthesize 5-HT (8). Peripherally synthesized 5-HT acts as a hormone molecule, whereas neuronally synthesized 5-HT acts as a neurotransmitter, and both are involved in cardiovascular function. metabolic disorders are closely associated with cardiovascular diseases, such as hypertension and atherosclerosis, as well as various conditions, including irritable bowel syndrome and depression (63, 64).

### 2.3 Indole pathway

The gut microbiota can work together to convert Tryptophan into indole and its derivatives, such as indole-3-propionic acid (IPA), indole-3-acetic acid (IAA), indole-3-aldehyde (IAID), etc (65). (Figure 1). After entering the liver, indole is hydroxylated by hepatic cytochrome P450 (CYP2E1) to form 3-hydroxyindole, which is then catalyzed by sulfotransferase (SULT) to form indoxyl sulfate (IS) (66). Preclinical studies have confirmed that IS, as a uremic toxin, promotes reactive oxygen species (ROS) production by activating NADPH oxidase, enhances oxidative modification of low-density lipoprotein (LDL), drives macrophage polarization toward the pro-inflammatory M1 phenotype, and promotes foam cell formation, thereby exacerbating CVDs and atherosclerotic plaque formation in chronic kidney disease (67, 68). Conversely, gut microbiota-derived indole derivatives such as IPA and IAID exert cardiovascular protective effects (5). Clinical studies have shown that indole metabolites (including indole, IPA, and IAID) are significantly and negatively correlated with the severity of advanced atherosclerosis (69). Mechanistically, IPA promotes reverse cholesterol transport in macrophages via the SPI1/miR-142–5p/ABCA1 pathway to attenuate atherosclerosis, while simultaneously activating the AKT signaling pathway to inhibit endothelial cell apoptosis, reduce mitochondrial ROS generation, and downregulate pro-inflammatory factors (TNF-α, IL-1β, IL-6), thereby maintaining endothelial tight junction integrity and delaying the progression of aortic dissection (AD) (70, 71). Meanwhile, IAID reduces endothelial ROS levels and suppresses IL-6 expression via the AhR/NRF2/HO-1 pathway to delay atherosclerosis, and also reduces macrophage infiltration, inhibits vascular smooth muscle cell (VSMC) phenotypic switching and extracellular matrix (ECM) degradation, thereby attenuating AD progression (72, 73).

### 2.4 The aryl hydrocarbon receptor

The aryl hydrocarbon receptor (AhR) is a ligand-dependent transcription factor that was initially identified as a receptor for heterocyclic toxins, such as dioxins, and has long been a subject of toxicological research (74). Currently, the physiological effects of AhR have been expanded to include immune response regulation, organogenesis, tumors, mucosal barrier function, cell cycle, and other aspects, and AhR is indispensable for maintaining cardiovascular homeostasis (75). In the cardiovascular system, AhR signaling plays an important role in maintaining cardiac function, development, angiogenesis, and physiological homeostasis (76). Key metabolites of KP and indole pathway metabolites formed by the gut microbiota are important endogenous AhR ligands. Preclinical evidence suggests that imbalances in KP metabolism lead to abnormal activation of AhR, which in turn triggers signaling through classical or non-classical pathways, inducing the expression of numerous inflammatory factors. These factors participate in pathological processes, such as immune-inflammatory responses, oxidative stress, endothelial dysfunction, and myocardial fibrosis, and are closely associated with the onset and progression of cardiovascular diseases (77). However, the causal association needs to be further confirmed by human studies.

## 3 The role and metabolic characteristics of KP in various CVDs

The role of KP metabolites in CVDs is complex and multifaceted. Animal experiments proved that they are deeply involved in the pathogenesis of various cardiovascular disorders, including myocarditis, heart failure, and atherosclerosis by modulating immune responses, oxidative stress, endothelial dysfunction, and myocardial hypertrophy. Notably, population-based studies have revealed that plasma or tissue concentrations of KYN and its downstream products closely correlate with disease severity, progression rate, and clinical endpoints, positioning them as promising novel biomarkers for early diagnosis and risk stratification, as well as innovative therapeutic targets. This section will systematically synthesize evidence on altered metabolites of KP levels and aberrant expression of key enzymes across various CVDs, and critically evaluate their translational potential and prognostic value.

### 3.1 Myocarditis, cardiomyopathy

Myocarditis can be triggered by various factors, with viral infections being the most common cause (78). Viral myocarditis (VMC) is a chronic life-threatening disease that typically manifests as a severe inflammatory response, myocardial damage, ventricular remodeling, and fibrosis, and can progress to cardiomyopathy (79). Studies suggest that inhibiting IDO may reduce the inflammatory response in macrophages, thereby alleviating myocardial damage in VMC mice (80). Notably, KP imbalance may serve as a common mechanism linking different etiologies to myocardial injury.

Firstly, a Mendelian randomization study has provided causal evidence at the genetic level that elevated KYN is a causal risk factor for myocarditis (OR = 1.383, 95%CI = 1.102–1.738, *p* = 0.005), which provides valuable guidance for early screening, prevention, and treatment of myocarditis (4). Second, in patients with chronic Chagas cardiomyopathy (CCC) induced by *Trypanosoma cruzi* infection, the KP is activated but severely imbalanced. Its metabolite profile is characterized by elevated levels of toxic metabolites, such as KYN and QA, along with decreased levels of protective branch metabolites including KYNA and xanthurenic acid XA (Table 2). This imbalance, together with decreased AhR activity, collectively leads to the failure of anti-inflammatory regulation and triggers a pro-inflammatory cytokine storm (e.g., IL-6 and TNF-α), ultimately exacerbating myocardial inflammatory injury and fibrosis (81). Not only that, KP imbalance is also a key link in non-infectious myocardial injury. Environmental radiation can also disrupt KP in hearts, increase KYN metabolite levels, cause oxidative stress and inflammation, and eventually lead to radiation cardiomyopathy (110).

**Table 2:** Characteristics of KP metabolism in CVDs.

Type of CVDs	Origin of samples	Method of detection	Number of participants	Metabolites, Enzymes and receptor of KP	References
CCC	Plasma	LC-MS	*n* = 47	QA, KYN↑;	(81)
				KYNA, XA↓, AhR↓	
HF	Plasma	LC-MS	*n* = 1211	KYN, 3-HK, QA, KTR, 3-HK/XA↑	(82)
HF in CKD	Serum	HPLC	*n* = 673	KYN, KTR↑; Trp↓	(83)
HFpEF and T2D	Serum	HPLC	*n* = 120	Trp, KYN, AA↑	(84)
				3-HK, QA↓	
CHF	Plasma	LC-MS	*n* = 3150	KTR↑	(12)
HF with RME	Serum	LC-MS	*n* = 55	KYN↑	(85)
HFrEF	Pectoralis major muscle	LC-MS	*n* = 28	KYN↓, KYNA↑	(86)
Early AS	Serum	HPLC	*n* = 986	Trp, KYN, KTR↑	(87)
Advanced AS	Serum	HPLC	*n* = 921	Trp, KYN, KTR↑	(88)
AS	Plaque-containing aortic tissue	UHPLC/TQ MS	*n* = 42	Trp, KYN, KTR, QA↑	(89)
AS	Carotid plaques	HPLC	*n* = 20	KYNA↑	(90)
AMI	Plasma	HPLC	*n* = 981	Trp, KYNA↑	(90)
AS in HIV	Plasma	HPLC	*n* = 737	Trp↓	(91)
				KYNA, KYNA/Trp↑	
Instability of AS	Serum	ELISA	*n* = 72	KMO↑	(92)
AAA	Aortic tissue	CE-TOFMS	*n* = 52	Trp, KYN, KTR, QA↑	(93)
				KYNU, KMO↑	
MIS	Plasma	LC-MS	*n* = 2819/2731	KYN, KTR, 3-HK↑	(94, 95)
Stable CAD	Urine	GC-MS	*n* = 3224	KTR↑	(96)
Stable CAD	Plasma	LC-MS	*n* = 2380	KTR↑	(97)
Stable CAD	Plasma	LC-MS	*n* = 4122	KTR, KYNA, 3-HK↑	(98)
				AA, 3-HAA↑	
Stable CAD	Serum	HPLC-MS	*n* = 170	Trp↓, IDO↑	(99)
CHD	Urine	UHPLC- MS	*n* = 550	Trp↑	(100)
CAD	Serum	HPLC-MS	*n* = 2970	Trp↓	(101)
LV remodeling of CAD	Plasma	UHPLC- MS	*n* = 1606	KYN, KYNA↑	(102)
Acute STEMI	Plasma	LC‒MS	*n* = 173	AA, Trp↑	(103)
				XA, 3-HK↓	
Early MI	Plasma	UHPLC- MS	*n* = 46	Trp↓	(104)
Significant CAD	Plasma	HPLC-MS	*n* = 305	KYN, IDO↑	(105)
IPAH	Plasma	HPLC-MS	*n* = 37, *n* = 60	KYN, Trp↑	(106, 107)
RV-PV dysfunction	Plasma	LC-MS	*n* = 71	KYN, KYNA↑	(108)
				AA, QA↑	
PAH	Plasma	HPLC	*n* = 56	Trp, KYN, KTR↑	(109)
				KYNA/KYN, AA/ KYN↓	
				3-HK/KYN↓	

CCC, chronic Chagas cardiomyopathy; HF, Heart failure; CKD, chronic kidney disease; HFpEF and T2D, preserved ejection fraction combined with type 2 diabetes; RME, reduced muscle endurance; HFrEF, heart failure with reduced ejection fraction; AS, Atherosclerosis; AMI, acute myocardial ischemia; AAA, Aortic Atherosclerotic Aneurysm; CAD, coronary artery disease; CHD, Coronary heart disease; STEMI, ST-segment elevation myocardial infarction; IPAH, idiopathic PAH; PAH, Pulmonary arterial hypertension; RV-PV dysfunction, right ventricular-pulmonary vascular (RV-PV) dysfunction; GC-MS, gas chromatography coupled with tandem mass spectrometry; HPLC, high-performance liquid chromatography; UHPLC-MS, ultrahigh-performance liquid chromatography with tandem mass spectrometry; HPLC-MS, high-performance liquid chromatography with tandem mass spectrometry; ELISA, enzyme-linked immunosorbent assay. ↑ increase, ↓ decrease; 3-HAA, 3- hydroxyanthranilic acid; 3-HK, 3-hydroxykynurenine; AA, anthranilic acid; KTR, Kynurenine/tryptophan ratio; KYN, Kynurenine; KYNA, Kynurenic acid; QA, quinolinic acid; Trp, tryptophan.

### 3.2 Heart failure

Heart failure (HF) is a complex clinical syndrome with heterogeneous pathophysiology and multifaceted etiology (111). Clinical studies have shown that KP is not only significantly dysregulated in HF patients with subtype specificity (Table 2), but also connects immune activation with myocardial metabolic remodeling, which is involved in the key pathophysiological link of the occurrence and development of HF.

**Figure 2:**
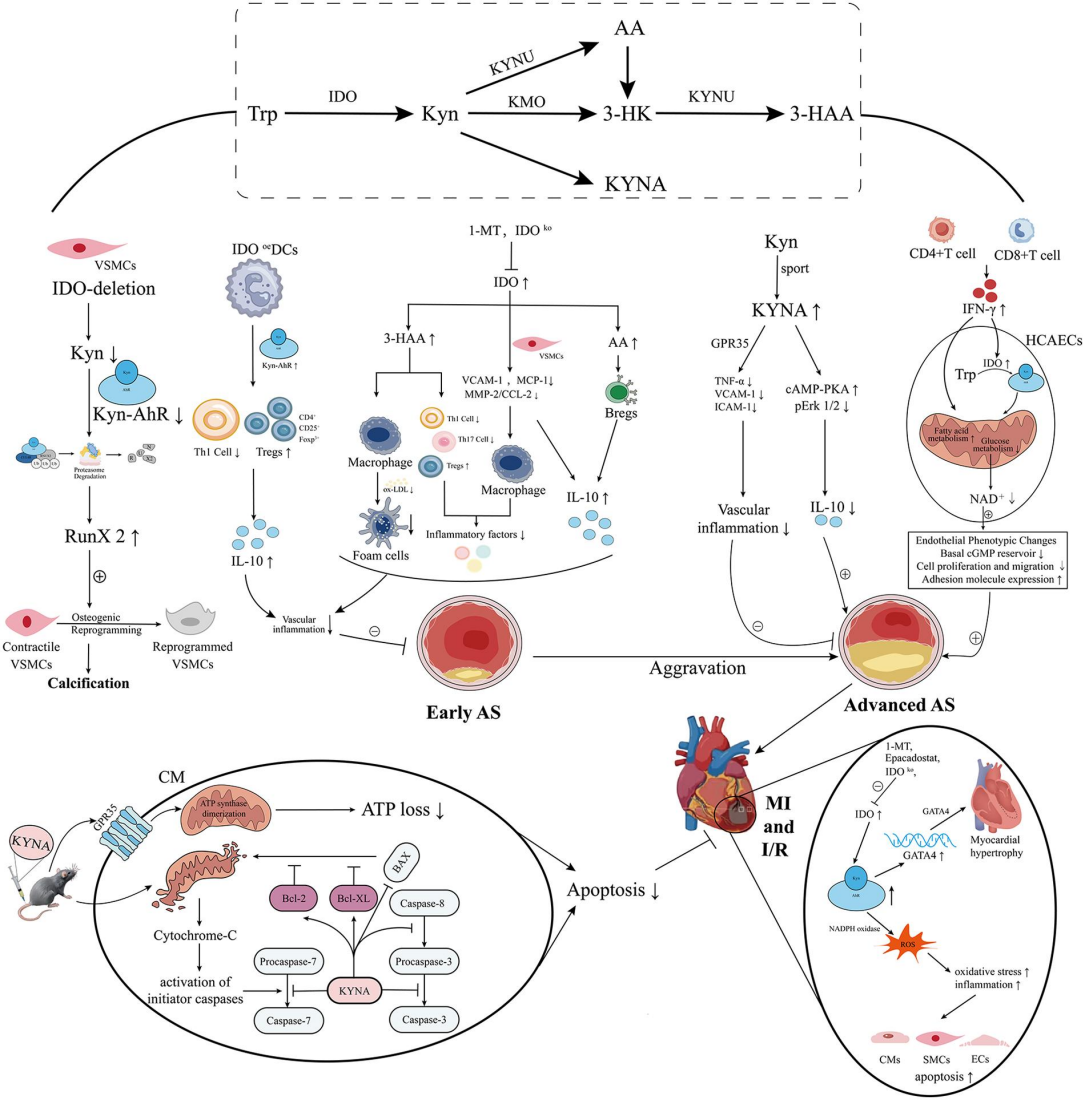
Elaboration of KP mechanisms in various CVDs (see text for details). VSMCs, Vascular smooth muscle cells; HCAECs, human coronary artery endothelial cells; CM, cardiomyocytes; SMCs, smooth muscle cells; ECs, endothelial cells; Tregs, regulatory T cells; Bregs, regulatory B cells; RUNX2, Runt-Related Transcription Factor 2; IDO^oe^ DCs, Dendritic cell-expressed IDO; ox-LDL, oxidized low-density lipoprotein; MI and I/R, Myocardial ischemia and ischemia-reperfusion; AS, atherosclerosis; VCAM-1, vascular cell adhesion molecule-1; MCP-1, monocyte chemotactic protein-1; GPR35, G-protein coupled receptor 35; 1-MT,1-methyl tryptophan.

The kynurenine-to-tryptophan (KYN/Trp, KTR) ratio serves as a marker of IDO activity, and elevated IDO activity is a hallmark of chronic inflammatory states. In individuals at high cardiovascular risk, elevated levels of KTR, KYNA, and QA prospectively predict the future risk of HF, offering novel blood-based indicators for primary prevention of CVDs (112). In the context of chronic kidney disease (CKD), KYN and KTR increase progressively with advancing CKD stage, correlate significantly with prevalent heart failure, and KTR serves as a strong predictor of HF risk in CKD patients (*p* = 0.019) (83). Upon progression to chronic HF, KTR further increases and correlates positively with HF severity—reflected by functional class and reduced left ventricular ejection fraction—and independently predicts the risk of cardiovascular death and cardiac transplantation. Even among HF patients with low-grade inflammation and normal hs-CRP levels (≤6 mg/L), elevated KTR can still identify a high-risk subgroup, demonstrating complementary prognostic value (12). Another study found that in HF patients, levels of Kyn, 3-HK, QA, and the ratios KTR and 3-HK/XA were significantly elevated, with 3-HK/XA (hazard ratio = 1.67) emerging as an independent risk factor for all-cause mortality. Notably, KYN exhibited superior predictive performance for mortality compared with the traditional gold-standard HF biomarker, NT-proBNP (82). In the HFpEF subtype, the KP profile is characterized by high Trp and KYN levels. Patients with concomitant type 2 diabetes exhibit more pronounced KP dysregulation, showing higher serum levels of Trp, KYN, and AA, but lower levels of 3-HK and QA. Moreover, KYN levels show a positive correlation trend with cardiac hypertrophy and dysfunction (84). Animal experiments have demonstrated that sustained high expression of IDO1 in cardiac tissue promotes pathological myocardial hypertrophy and fibrosis via the KYN-AhR-GATA4 signaling cascade, indicating that KYN actively contributes to cardiac remodeling in HF and represents a potential therapeutic target (113) (Figure 2). Furthermore, KYN is significantly elevated in HFrEF patients with reduced muscular endurance (RME). It can independently distinguish the presence of RME with 83% accuracy (AUC = 0.83), providing a simple screening tool for identifying severe skeletal muscle dysfunction in HF (85). Research indicates that sodium-glucose cotransporter 2 inhibitors (SGLT2i) alleviate skeletal muscle atrophy in HFrEF patients, thereby markedly improving clinical outcomes. This benefit is mediated by a reduction in serum IL-6, which directly suppresses IDO 1, leading to decreased KYN production and, consequently, to diminished expression of muscle atrophy-related genes (MuRF1) (86).

In summary, KP metabolites and their ratios not only provide early warning of incident HF but also enable refined stratification of mortality and hospitalization risk, identification of skeletal muscle dysfunction, and reflection of subtype-specific metabolic remodeling in HF. This positions the KP pathway as a promising next-generation biomarker for multiple clinical scenarios and a potential therapeutic target.

### 3.3 Atherosclerosis

Atherosclerosis (AS) is a chronic inflammatory disease that primarily affects the inner layer of the blood vessel wall. It is characterized by lipid deposition beneath the endothelium, foam cell formation, and migration and proliferation of vascular smooth muscle cells (114). Immune inflammation and metabolic disorders are important pathogenic mechanisms in AS; however, no effective therapies have been developed to date (115). KP can participate in AS through a dual complex regulatory mechanism involving immunity and metabolism; however, the metabolic characteristics of KP and its role it plays will differ at different stages of AS.

Early AS is characterized by increased carotid intima-media thickness (c-IMT). In two studies involving young and middle-aged individuals, enhanced serum IDO activity was associated with increased c-IMT and multiple cardiovascular risk factors, including age, BMI, plasma lipids, and CRP. These findings suggest that IDO enzymes may be involved in the immune regulation of early AS and could serve as potential novel biomarkers for immune activation in early AS in women (87, 88). Subsequent studies have further confirmed that IDO1 exerts a protective effect in early AS by modulating immune-inflammatory responses. In the ApoE−/− knockout mouse model, deficiency of IDO1 or the use of its specific inhibitor 1-methyltryptophan (1-MT) upregulates the expression of pro-inflammatory factors such as vascular cell adhesion molecule-1 (VCAM-1) and monocyte chemoattractant protein-1 (MCP-1). This promotes the infiltration and accumulation of CD68^+^ macrophages within plaques while downregulating the expression of the anti-inflammatory cytokine IL-10. As a result, vascular inflammation is aggravated, accelerating the progression of early AS (116–118). Similarly, in animal models of non-alcoholic steatohepatitis combined with AS, deficiency of IDO1 also worsens atherosclerotic lesions (119). Mechanistically, IDO1 can regulate vascular smooth muscle cells (VSMCs) through the KYN-AhR-cullin 4B-RUNX2 signaling axis, preventing arterial calcification (120). Overexpression of IDO1 in plasmacytoid dendritic cells in the aorta increases aortic KYN concentration and enhances AhR activation. This promotes the expansion of local CD4^+^CD25^+^Foxp3^+^ regulatory T cells (Tregs) and strengthens IL-10 expression, exerting an immunosuppressive effect. This process reduces Th1 cell infiltration and vascular inflammation, ultimately leading to a significant reduction in aortic plaque area and demonstrating clear anti-atherosclerotic activity (121, 122). In addition, other KP metabolites exert protective effects. AA significantly increases the production of IL-10 by B cells. 3-HAA inhibits proinflammatory Th (1, 2, 17), increases the proportion of Tregs, and controls the SREBP/lipoprotein axis in the liver, inhibiting inflammasome activation in macrophages and their uptake of oxidized low-density lipoprotein (Ox-LDL) (123, 124). Both reduced local inflammation in the blood vessels.

With the progression of AS, although KYN, Trp, KTR, QA were significantly increased in plaque tissue (89), plasma KP metabolites were associated with the progression of AS at this time (Table 2). Clinical studies have confirmed that KYNA is enriched in unstable plaques, and its plasma concentration is significantly correlated with plaque instability, making it a potential biomarker for evaluating the progression and prognosis of AS (90). This finding was further corroborated by people living with HIV. Lower plasma Trp level, higher plasma KYNA level and its ratio (KYNA/Trp) can independently predict the risk of carotid plaque progression in HIV patients (91). These findings collectively suggest that the degree of activation of the KP metabolic pathway, AS reflected by the KYNA/Trp ratio, is not only an indicator of AS activity, but also potentially a novel biomarker for predicting the progression of AS, including HIV-associated AS. At the mechanistic level, KYNA was found to promote the progression of AS through a dual pathway: on the one hand, activation of camp-dependent pathway and on the other hand, inhibition of Erk1/2 phosphorylation, which jointly lead to a decrease in the level of anti-inflammatory factor IL-10. Additionally, other studies have found that KMO expression levels are significantly increased in unstable plaques and are closely related to plaque instability. Moreover, KMO expression level was positively correlated with the promoting M0 macrophages and negatively correlated with the protecting CD8 + T cells. Experiments showed that silencing KMO attenuated plaque formation and promoted stability in ApoE-/- mice (92, 93). Of note, KP also acts through other mechanisms to accelerate AS progression: secretion of IFN-*γ* by intraplaque T lymphocytes, up-regulation of IDO activity in human coronary endothelial cells, and accelerated metabolism of tryptophan to KYN. This activates AhR, inhibits HIF-1*α* stability, impairs glycolysis, impairs glucose metabolism, and consistently consumes NAD+, further driving a metabolic shift toward fatty acid oxidation. Ultimately, these metabolic reprogramming events lead to adverse alterations in coronary endothelial phenotypes, including reduced basal cGMP levels, impaired endothelial migratory capacity, and accelerated proinflammatory transitions, which contribute to AS progression (125).

The KP plays a dual and dynamic role in AS. In early disease stages, IDO1 activation expands Tregs and increases anti-inflammatory factors, offering protection. However, in late stages with constant inflammation, KP function changes and worsens the disease. Ongoing IDO1 activity disrupts macrophage cholesterol processing, causing lipid buildup. Also, enzymes like KMO are increased in unstable plaques. This leads to more pro-inflammatory metabolites (e.g., 3-HK, QA) and more pro-inflammatory M0 macrophages, together making plaques less stable. This shift happens because of changes in the immune environment, cell types, and metabolism. Therefore, the plasma KTR and KYNA level are potential biomarkers for body-wide inflammation and plaque risk. Accordingly, IDO1 and KMO are promising treatment targets. However, their effects depend heavily on the disease stage and cell context. The exact mechanism driving this functional shift remains unclear. Achieving precise treatment is a key challenge for future research.

### 3.4 Myocardial ischemia syndrome

Myocardial ischemia syndrome, including ST-segment elevation myocardial infarction, non-ST-segment elevation myocardial infarction, stable angina, and unstable angina, has a high incidence and mortality rate worldwide (126). KP metabolites exhibit dynamic changes in various types of myocardial ischemia syndromes. The Holland Health Study identified elevated plasma KTR, KYN, and 3-HK concentrations as important predictors of acute coronary events or mortality in elderly patients without coronary artery disease (CAD) (Table 2), suggesting that they may serve as potential biomarkers for CAD risk stratification (94, 95). Several studies on patients with stable angina (SA) have revealed that elevated levels of KTR in blood or urine, decreased levels of Tryptophan in plasma, and elevated levels of KP metabolites (KYN, 3-HK, AA, and 3-HAA) can predict coronary events and all-cause mortality in SA patients (96–99). Three studies on patients with CAD in China found similar results (100–102).

In the stage of acute myocardial infarction, the metabolite profiles show different characteristics. In an analysis of patients with acute ST-segment elevation myocardial infarction found increased levels of AA and Trp and decreased levels of XA and 3-HK (103). In contrast, another study observed a decrease in serum tryptophan metabolism within the first 5 min after early myocardial ischemia (MI) in humans (104). In addition, elevated KYNA levels in the blood indicate a high risk of acute MI, and KYNA is currently being studied as a potential biomarker for CAD (127). There is academic controversy about the role of IDO. Wongpraparut et al. found that its activity is significantly related to the number and severity of coronary stenosis, and it can be used as an marker driven by inflammation (105). In contrast, a Mendelian randomization study showed that high IDO1 protein levels are negatively correlated with the risk of ischemic heart disease (IHD), suggesting that it exerts a protective effect through anti-inflammatory or immune regulatory mechanisms (128). In terms of diagnosis, indicators related to KP have shown breakthrough potential. In distinguishing between acute coronary syndrome (ACS) and SA, KMO has shown superior diagnostic efficacy to hs-CRP, with higher sensitivity and specificity (92). Another study explored new ACS biomarkers and identified three plasma metabolites—lysine, isocitrate, and tryptophan—whose combined area under the receiver operating characteristic curve was 1.00, making them more effective than troponin I and CK-MB in distinguishing ACS from healthy controls (129).

Experimental studies on pathological mechanisms reveal the complex role of this pathway. Melhem et al. found that up-regulated IDO1 after MI can significantly induce KYN production, activate AhR, induce NADPH oxidase, and increase ROS production, and ultimately lead to apoptosis of smooth muscle cells, endothelial cells, and cardiomyocytes (130). In contrast, in non-ST-segment elevation acute MI, IDO1 expression can suppress T cell proliferation and apoptosis to alleviate inflammatory responses (131). Regarding the therapeutic potential of KP metabolites, the role of KYNA in cardioprotection is supported by preclinical evidence. For instance, animal experiments demonstrate that injection of KYNA can significantly reduce myocardial infarct size (132). Further study reveals that KYNA protects cardiomyocytes from ischemia-reperfusion (I/R) injury by activating GPR35 receptor, mediating mitochondrial ATP synthase dimerization to conserve myocardial energy, and exerting a significant anti-apoptosis effect (133). However, it is critical to contextualize these findings:​ the evidence remains preliminary as it is derived solely from animal models, and human trial data are currently lacking. Furthermore, safety concerns must be addressed, as KYNA interacts with multiple receptors (e.g., AhR, α7nAChR, ionotropic glutamate receptors) beyond GPR35, raising the risk of off-target effects. Therefore, the translation of KYNA-based strategies requires caution, and future work should focus on validating these mechanisms in human studies and developing selective agonists to minimize non-targeted risks.

In terms of treatment, part of the benefits of exercise training as a non-pharmacological intervention are thought to be achieved by regulating this pathway. Animal experiments proved that exercise can promote the conversion of KYN with anti-inflammatory properties to KYNA, which inhibits the release of TNF-α by activating GPR35. At the same time, exercise can also reduce the level of soluble adhesion molecules to improve endothelial function and inflammation, thereby delaying the progression of CAD (134). In particular, experiments have shown that high-intensity interval training (HIIT) is more effective in inhibiting the IDO1-KYN-AhR axis, reducing oxidative stress and protecting the heart from MI (135). In addition, the cardioprotective effect of remote ischemic conditioning (RIC) was partially demonstrated to be dependent on the synthesis of KYN and its promotion of hepatic NAD+-dependent deacetylase sirtuin activity (136).

In summary, the KP permeates multiple dimensions of myocardial ischemic syndromes, encompassing risk prediction, disease diagnosis, pathological mechanisms, and therapeutic intervention. The dynamic alterations in its metabolites serve as pivotal biomarkers, while its intricate mechanistic network provides a crucial molecular rationale for developing stage-specific, precision cardioprotective strategies.

### 3.5 Pulmonary arterial hypertension

Pulmonary arterial hypertension (PAH) is a progressive, malignant cardiovascular disease characterized by significant remodeling of the pulmonary vascular system and a progressive increase in pulmonary vascular load, often leading to right heart failure and death in the advanced stages (137). Currently, treatment options are limited, and there is an urgent need for novel interventions that target the underlying pathophysiological mechanisms of PAH. KP is a promising option that should be considered.

The therapeutic potential of KP is manifested in both acute and chronic levels. In the short term, KYN can act as an acute pulmonary vasodilator, rapidly inducing vasodilation by activating guanylyl cyclase and adenylate cyclase in pulmonary artery smooth muscle cells (107). In the long term, enhancing the expression of rate-limiting enzyme IDO1 in pulmonary endothelial cells can reverse pathological vascular proliferation and regulate the phenotypic transformation of VSMC, thereby delaying pulmonary vascular remodeling from the root and providing sustained protection (138).

In addition to treatment, KP metabolites such as KYN, AA, and QA can also serve as specific non-invasive circulating biomarkers. Based on the analysis of Chuang Yang et al. and multiple studies, KP plays a central role in pulmonary arterial hypertension (PAH) and right ventricular-pulmonary vascular (RV-PV) dysfunction (106). The levels of Trp, KYN, KTR, and other metabolites (such as KYNA, AA, and QA) in the plasma of PAH patients were significantly elevated (109, 139). Elevated levels of these KP metabolites are directly associated with impaired right ventricular diastolic function, pulmonary artery-right ventricular decoupling, decreased right ventricular ejection fraction (RVEF), and reduced exercise capacity. Their levels are positively correlated with resting/exercise states and can independently predict outcomes, with a predictive value significantly superior to that of the traditional marker NT-proBNP (108, 140). Serum KYN showed a strong positive correlation with mean pulmonary artery pressure (mPAP), and the AUC for diagnosing PAH using serum KYN was as high as 0.86 (107).

In summary, the KP not only provides a potential therapeutic target for the intervention of PAH, from rapid symptom relief to long-term disease modification, but also its abnormal activation metabolic profile itself provides a reliable tool that is superior to traditional indicators for early identification, severity assessment and accurate prognosis management of the disease.

### 3.6 Hypertension

Hypertension (HTN) results from the combined effects of genetic and environmental factors and is a major risk factor for CVD, stroke, disability, and death. Over the past few decades, solid progress has been made in the understanding, control, and drug development of HTN; however, a significant proportion of patients with HTN still do not achieve an optimal response to antihypertensive drug therapy (141). However, KP can provide new strategies for preventing and treating hypertension.

The core metabolite of this pathway, KYN, is an endothelium-derived relaxing factor that regulates blood pressure. Wang et al. reported that under inflammatory conditions, upregulation of IDO1 in vascular endothelium increases KYN, which dilates blood vessels and lowers blood pressure by activating the cAMP/cGMP signaling pathway. Animal experiments have confirmed that intravenous infusion of KYN (0.15–1.5 mM) can lower blood pressure in spontaneously hypertensive mice in a dose-dependent manner (142). In addition, KYN can also activate voltage-dependent Kv 7 channels encoded by the KCNQ gene family in VSMC to lower blood pressure in rats (143). Subsequent studies have shown that KYN derived from IDO1 is a new mediator of hypotension in human sepsis, and that inhibition of IDO1 can alleviate septic hypotension and mortality (144). Excitingly, the Phase I clinical trial of KYN showed that healthy volunteers tolerated intravenous doses ranging from 50 µg/kg to 5 mg/kg without any adverse reactions (145). However, another study also found that long-term intravenous infusion of KYN, even with only a relatively small increase in serum KYN concentration (from 0.2 µM to 0.5 µM), would lead to deterioration of renal function and increase in mean arterial pressure in rats (146). This suggests that future research should focus on the safe duration and administration regimen of KYN application.

## 4 Research on treatment strategies targeting KP in CVDs

The KP plays an important role in human health and CVDs, and the regulation of kynurenine metabolism has clinical and therapeutic significance.

### 4.1 Development of enzyme inhibitors and agonists

Regulatory strategies targeting the KP, especially the bidirectional development of agonists and inhibitors, have therapeutic potential for CVDs. In terms of agonists, research shows that enhancing the activity of IDO1 has significant potential. For instance, dendritic cell vaccines with overexpressed IDO1 can induce the immunomodulatory effect of Tregs in the local vascular system of early lesions of AS, providing a new preventive strategy for immune intervention of AS (122). Similarly, specifically enhancing the expression of IDO1 in pulmonary endothelial cells has been proven to effectively reverse pathological vascular proliferation, regulate the VSMC phenotype, thereby alleviating vascular remodeling in PAH and exerting a long-term protective effect (138). These findings suggest that in specific early stages of diseases such AS and PAH, the IDO activation strategy is expected to exert therapeutic benefits by reshaping the immune microenvironment and inhibiting abnormal vascular remodeling.

The development of KP inhibitors, primarily targeting KMO and IDO1, has made significant progress. They have shown promise in preclinical models of CVDs, and some IDO1 inhibitors have even entered clinical evaluation for cancer immunotherapy. In cardiovascular preclinical studies, inhibiting KMO reduces inflammation and improves survival in VMC models (147), while its genetic silencing promotes atherosclerotic plaque stability (92). IDO1 inhibitors (e.g., 1-MT) can alleviate damage in chronic myocarditis and improve neurological outcomes after cardiac arrest (148). Currently, several highly selective KMO inhibitors (e.g., GSK180, GSK065, GSK366) have demonstrated organ-protective effects in animal models like acute pancreatitis (149, 150). Various IDO1 inhibitors (e.g., Epacadostat, PF-06840003) have also progressed to clinical trials in oncology. For instance, PF-06840003, an oral and highly selective IDO1 inhibitor, showed good tolerability and a 47% disease control rate in a Phase I trial for recurrent glioma, although its efficacy as a monotherapy was limited, necessitating further exploration in combination with immunotherapy (151). Epacadostat combined with a PD-1 antibody showed promising response rates in early trials but failed to improve progression-free survival in a pivotal Phase III study, leading to its discontinuation. Other related Phase III trials (in NSCLC, UC, HNSCC) were also terminated or are awaiting results (152, 153).

This setback offers a crucial lesson for translational research in CVDs. It highlights that the efficacy of IDO inhibitors is highly dependent on the specific disease's immune microenvironment and pathological context. Blindly transplanting strategies from one field carries significant risks. Therefore, future KP-targeted therapies in cardiology should first systematically study the spatiotemporal dynamics and tissue specificity of KP metabolism in specific CVDs models. It is essential to clarify its potentially dual regulatory roles at different disease stages—for example, its distinct functions in early vs. late AS, or in acute myocarditis vs. chronic myocarditis and subsequent HF. Second, biomarker-based patient stratification criteria should be established to enable precise intervention for subgroups with clearly aberrant KP activity. Finally, a phased validation strategy should be adopted in clinical translation: conducting proof-of-concept studies to confirm efficacy signals before proceeding to large-scale clinical trials. This mechanism-informed, patient-selection-focused, and stepwise development path provides a feasible framework for the successful translation of KP-targeted therapies in the field of CVDs.

### 4.2 Dietary intervention, KP metabolite supplements, and derivative development

Dietary intervention offers a new non-pharmacological strategy for treating hypertension and has been shown to exert its effects through the KP. A tryptophan-rich birdseed water extract (AEPc) increases KYN levels, which dilates blood vessels and lowers blood pressure via NO-dependent pathways. This effect is enhanced when combined with exercise (154). Recent studies have also shown that dietary taurine supplementation can target and regulate KP to lower blood pressure and protect the cardiovascular system by repairing gut-brain axis function and inhibiting inflammation (155).

In early AS, KYN supplementation can effectively inhibit VSMC calcification through a RUNX2-dependent mechanism, thereby achieving phenotypic rescue (120). The downstream metabolite of KP, 3-HAA, directly enhances the dual anti-inflammatory and lipid-regulating pathways during AS formation by controlling the hepatic SREBP/lipoprotein axis, inhibiting pro-inflammatory Th (1, 2, 17) cells, increasing the proportion of Tregs, inhibiting inflammatory body activation in macrophages and their uptake of oxidized low-density lipoprotein (Ox-LDL), and alleviating LDLr−/− mouse AS (123, 124). AA and its synthetic derivative 3,4-dimethoxycinnamoyl AA significantly increase the production of IL-10 by splenic B cells, alleviated arterial inflammation, and reduced proinflammatory cytokine secretion. Analogs such as quinisite (3,4-DAA) can downregulate the expression of inflammatory factors in patients with AS. Injection of KYNA into animals with myocardial infarction significantly reduces the infarct area and protects against myocardial ischemia (132). Therefore, strategies involving supplementation with KYN, KYNA, 3-HAA, and AA levels, or supplementation with analogs, may have high therapeutic potential in the prevention and treatment of atherosclerotic CVDs.

### 4.3 Medication or traditional Chinese medicine, gut microbiota intervention

As mentioned above, SGLT2i significantly improves myocardial and skeletal muscle dysfunction in HFrEF, thereby improving clinical outcomes. It has become a breakthrough treatment option for improving survival rates and quality of life in patients with HF. Sacubitril/valsartan, which is highly recognized as a drug for treating chronic heart failure (CHF), downregulates the mRNA and protein expression of IDO in the myocardial tissue of HF rats, exerting a cardioprotective effect by inhibiting KP (156). Therefore, existing drug-based KP intervention strategies can extend their protective effects against CVDs.

Developing traditional Chinese medicine to intervene in KP in cardiovascular disease is a valuable strategy. Aloe emodin (a single anthraquinone compound in traditional Chinese medicine) can alleviate radiation-induced cardiac damage. This is because it inhibits the accumulation of aspartate transaminase (GOT2) on damaged mitochondria to stabilize KP and reduce the production of KYN (110). Baicalin, a natural flavonoid and the main active ingredient in the traditional Chinese herb *Scutellaria baicalensis Georgi*, alleviates inflammatory responses and myocardial ischemia damage by inhibiting AhR (157).

Supplementing with selected microorganisms can restore the intestinal microbiota, reduce plasma KYN levels, and alleviate ventricular remodeling. An interesting study successfully established a novel *in vitro* drug screening model for intestinal bacteria, screening for Clostridium spore-producing bacteria from the intestinal microbiota. These bacteria metabolize tryptophan into beneficial metabolites that counteract CAD or prevent the production of harmful metabolites, and once equilibrium is achieved, KYN downstream remains unchanged (158). Another study also found that a gut microbiota-derived metabolite, KYN, activates AHR and its gene targets, reshaping young hearts under stress overload. This process can be regulated and prevented by constructing a probiotic suspension containing three strains (Bifidobacterium longum 913, Lactobacillus acidophilus 145, and Enterococcus faecalis ATCC 19433) (159).

## 5 Limitations and future prospects

Although we have recognized the importance of the tryptophan-kynurenine pathway in the development of various CVDs, a deeper mechanistic understanding and clinical translation still face several key limitations.

First, methodological constraints hinder mechanistic analysis. Current research primarily relies on blood tests, which only reflect the average systemic levels of metabolites and cannot capture their real-time concentration, spatial-temporal distribution, or functional state in local microenvironments such as vascular walls, myocardium, or arterial plaques. The lack of “in situ detection technology” has left critical questions unresolved: we cannot determine the precise local concentration thresholds at which KP metabolites shift from protective to damaging effects in specific lesion sites, nor can we fully clarify the exact mechanisms of key metabolites in the human body. Second, there are profound metabolic differences between animal models and human physiology. KP metabolism exhibits significant species specificity. For instance, the concentration required for KYNA to activate AhR can differ by thousands of times between mouse and human cells, with its potency for human receptors being much stronger than in mice. Dosing and mechanisms based on animal models and blood indicators are often unreliable for predicting human responses, directly contributing to the setbacks of some KP-targeting strategies in clinical trials. Finally, these fundamental limitations in basic research have collectively led to a severe lack of clinical translation evidence. There is a critical shortage of high-quality, prospective human data that directly link specific KP metabolic dynamics to clear cardiovascular clinical outcomes.

Therefore, future research should focus on developing and applying advanced *in situ* detection metabolomics techniques to visualize and quantify KP metabolism in models that better reflect human biology, such as humanized animal models, human primary cells or induced pluripotent stem cell (iPSC)-derived systems, patient-derived organoids, thereby clarifying its pathological mechanisms. On this basis, a biomarker system capable of distinguishing disease stages and patient subtypes should be established.

**Figure 3:**
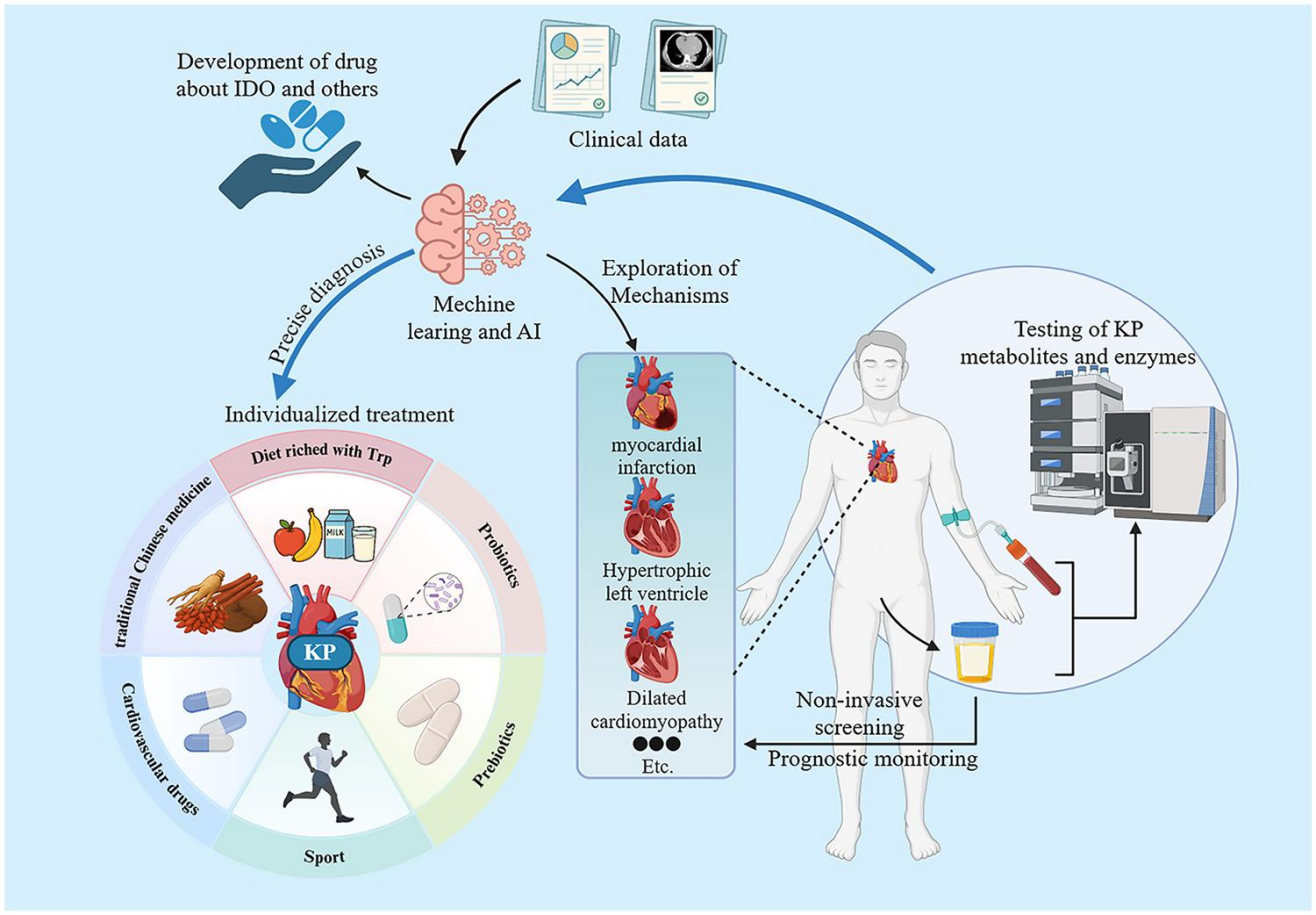
Research on treatment strategies targeting KP in CVDs and future prospects.

In order to overcome these limitations, metabolomics technology itself is undergoing a dual evolution from “holistic analysis” to “spatial resolution” and “clinical convenience”. Traditionally, KP metabolites in body fluids have been used as “molecular Windows” to characterize cardiometabolic disorders. However, it cannot resolve cellular heterogeneity and spatial microenvironment, which limits the in-depth understanding of the mechanisms of cell interactions in the microenvironment. Recently, single-cell and spatially resolved metabolomics technologies are filling this gap. By integrating imaging mass spectrometry (IMC) and matrix-assisted laser desorption ionization mass spectrometry (MALDI-MSI), we were able to simultaneously analyze immunophenotypes and metabolite profiles at the single cell level on the same tissue section (160). This spatial omics approach is expected to directly visualize the KP metabolic status (such as tryptophan depletion in the active region of IDO1) of specific cell populations (such as macrophages and T cells) in pathological tissues such as myocardial infarction or atherosclerotic plaques, so as to elucidate the formation mechanism of immunometabolic microenvironment and its specific role in disease progression in the spatial context. At the clinical translational level, the development of non-invasive urine detection techniques that overcome the limitations of clinical sample acquisition has enabled routine and efficient quantification of KP metabolites (161) (Figure 3).

Facing the resulting massive and high-dimensional data, machine learning (ML) and artificial intelligence (AI) have become indispensable analytical tools (162). In the field of metabolomics data analysis, ML technologies significantly enhance analytical capabilities, promoting their application in disease diagnosis, mechanism research, and clinical translation (163). For instance, Huang et al. utilized a deep learning network to develop a serum metabolite fingerprint diagnosis platform for breast cancer, achieving an area under the curve (AUC) of 0.948 and enabling efficient diagnosis (164). Meanwhile, an increasing number of researchers are integrating multi-omics data through algorithms such as DIABLO and MOTA, which synchronously integrate metabolomic, transcriptomic, and proteomic data to identify cross-omics biomarker panels (163). In the field of clinical risk prediction, Wenzl et al. used the XGBoost algorithm to develop the GRACE 3.0 scoring system for risk stratification in patients with non-ST-segment elevation acute coronary syndrome (NSTE-ACS). This model was developed based on large-scale cohort data from the UK and Switzerland (training set *n* = 386,591; external validation set *n* = 20,727) and specifically evaluated for its sex-specific performance, significantly improving risk stratification accuracy in female patients (165). In the field of drug discovery, AI technologies are also systematically reshaping the drug-discovery paradigm (166). Through virtual screening, deep generative models for designing small molecules (e.g., analogs of KP metabolites), and virtual models for predicting clinical trial outcomes, AI can significantly improve R&D efficiency, reduce failure rates, and accelerate drug development for metabolic targets such as IDO and KMO. For example, Chenthamarakshan et al. developed a deep learning generative foundation model (CogMol) that successfully designed novel inhibitors targeting key SARS-CoV-2 proteins using only target protein sequences (without relying on known active molecules or 3D structural information), achieving an experimental validation success rate of 50% and reducing the development cycle to several weeks (167). This case demonstrates that AI-driven generative methods not only reduce R&D failure rates but also provide efficient discovery pathways for targets lacking structural information. In summary, AI is likely to profoundly transform medical practice, making healthcare more precise and efficient.

## 6 Conclusion

The tryptophan-kynurenine pathway plays an important regulatory role in the occurrence and evolution of CVDs through its key enzymes and metabolites. They are not only potential biomarkers, but also the core regulators driving the disease process, and are also therapeutic targets. However, its effects show a “duality” and environmental dependence, that is, the same component can exert opposite protective or damaging effects in different disease stages or microenvironments. Although the specific mechanisms of KP in the pathophysiological processes such as immune response and oxidative stress are still controversial, these controversies point to the focus of future research: to deeply analyze the precise network of KP in specific cardiovascular pathological scenarios. At present, the in-depth research on KP is promoting cardiovascular medicine to precision from two levels of mechanism understanding and clinical transformation. Looking forward, the integration of new technologies such as single-cell spatial metabolomics and AI will be the key to breakthrough. The former can analyze the spatial heterogeneity of KP in diseased tissue, while the latter can integrate multi-dimensional data to construct a prediction model, and jointly promote the realization of a complete management closed-loop from “mechanism analysis, accurate diagnosis, targeted intervention, and dynamic monitoring”. Continuing to deepen the exploration of the KP pathway will open up new treatment opportunities and precision era for the prevention and control of CVDs.
